# ﻿Predicting landscape disturbance using adult Trichoptera: one (caddis) metric to rule them all?

**DOI:** 10.3897/zookeys.1263.141377

**Published:** 2025-12-10

**Authors:** David C. Houghton

**Affiliations:** 1 Department of Biology, Hillsdale College, 33 East College Street, Hillsdale, MI 49242, USA Hillsdale College Hillsdale United States of America

**Keywords:** Biomonitoring, caddisflies, diversity, richness, taxonomic resolution, ultraviolet light

## Abstract

The adult stage of Trichoptera is valuable for assessing the biotic integrity of streams; however, it is not clear which specific metric(s) have the greatest value for doing so. In this study, >500,000 adult caddisfly specimens reflecting 299 species were sampled and identified from 903 stream sites throughout the northcentral United States. Specimen data were compiled into 31 water quality metrics encompassing taxonomic richness, diversity indices, pollution tolerance, percent dominant taxa, and relative abundance of functional feeding groups. Each metric was individually tested for its ability to predict the known percentage of undisturbed habitat upstream of each sampling site using simple linear regression modeling. Most metrics were statistically significant but had *R*^2^ values <0.30. The highest performing models were taxonomic richness at the species (*R*^2^ = 0.40), genus (0.52), and family (0.59) levels and the Hilsenhoff Biotic Index (HBI) (0.37). The family level of taxonomic resolution produced models with a higher *R*^2^ value than genus or species for all four of the metrics where taxonomic resolution was tested. Multiple linear regression models of all 31 metrics (*R*^2^ = 0.65) and of combined family richness, HBI, and the ratio of shredders to filtering collectors (0.62) exhibited modest improvements over using family richness exclusively. These results indicated that simple taxonomic richness metrics constitute the most effective predictors of undisturbed upstream habitat when using adult caddisfly data, and that family richness may be the most effective of all due to low stochastic variation and ease of use.

## ﻿Introduction

Despite its ubiquity in biomonitoring protocols, several inherent problems decrease the effectiveness of the benthic life stage in assessing riverine biotic integrity. The success of sampling benthic aquatic insects is highly dependent on sampling effort (Cao et al. 2002) and the specific sampling device used. Many common benthic collecting devices such as kick nets, Hess samplers, and Surber samplers over-emphasize shallow habitats with uniform substrate, while under-sampling deep water, large woody debris, the undersides of large boulders, near-shore and bank habitats, and the entire hyporheic zone (Gerth and Herily 2006; Chessman et al. 2007; Cao and Hawkins 2011). Most larvae are not identifiable to the species level, and this lack of taxonomic resolution may result in a loss of information and decreased metric sensitivity (Hawkins et al. 2000; Stribling et al. 2008; Houghton et al. 2011). Furthermore, any benthic specimen that is incapable of surviving to adulthood in a specific stream, yet collected as a subterminal larva, would yield erroneous data about stream conditions. Only emergent adults can confirm the ability of a species to complete its entire life cycle in a particular habitat. Thus, while benthic sampling is a valuable technique for stream bioassessment (Barbour et al. 1999), it is possible that sampling the winged adult stage may be even more representative of stream conditions.

Caddisflies are a particularly important taxon for assessing riverine biotic integrity due to their high species richness, representative ecological diversity, and robust response to various anthropogenic disturbances (Barbour et al. 1999; Dohet 2002; Malm et al. 2013; Morse et al. 2019; Houghton and DeWalt 2023). Sampling the winged adult stage is particularly useful since most Nearctic specimens are identifiable to the species level and are attracted to ultraviolet lights regardless of their specific natal microhabitat (Houghton 2004). Moreover, while the exact area sampled by an ultraviolet light is not known, it almost certainly is greater than that of multiple types of benthic samples while simultaneously requiring less effort to obtain.

Since adult caddisflies are not yet frequently used in biomonitoring protocols, it is unclear if common water quality metrics will be effective when compiled using adult caddisfly data, or if certain metrics are superior to others. Therefore, the primary objective of this study was to test the ability of common metrics, both individually and collectively, to predict a known level of habitat disturbance. A secondary objective was to test the importance of taxonomic resolution (family, genus, or species) when compiling the various metrics.

## ﻿Material and methods

**Figure 1.**
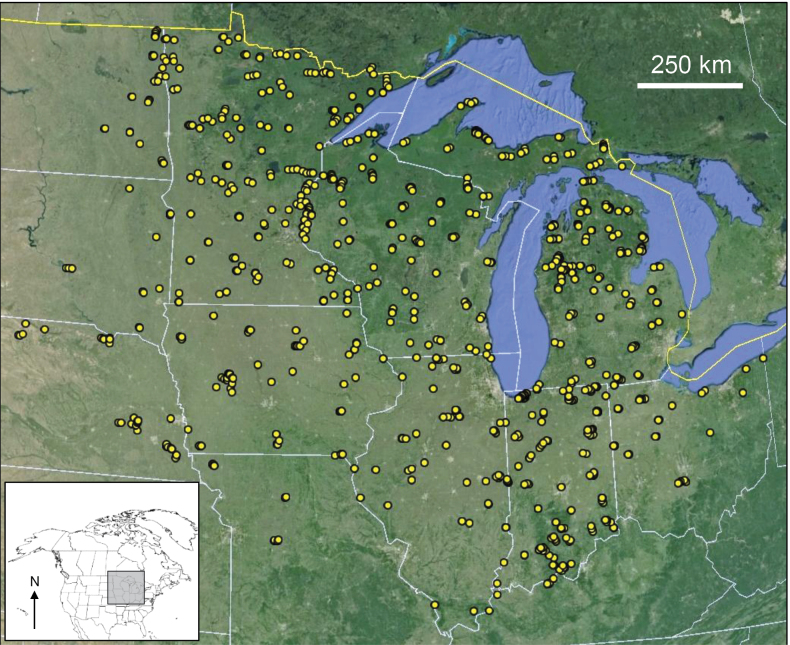
The 903 ultraviolet blacklight samples collected during this study throughout the northcentral United States. Substantial marker overlap occurs at this level of resolution. Base map © Google, NOAA.

Sampling began in 1999 and finished in 2022. During that period, 903 sites were sampled throughout an area approximating the Upper Midwest and Temperate Plains ecoregions of the northcentral United States (Omernik and Griffith 2014), and encompassing ~1.5 million km^2^ (Fig. 1). Large rivers such as the Mississippi were sometimes sampled at multiple locations, with each location considered a unique sampling site. The primary goal in choosing sampling sites was to cover as much area as possible with a consistent effort.

The land use of each of the 903 sample sites was determined using the United States Environmental Protection Agency (USEPA) StreamCat database (https://www.epa.gov/national-aquatic-resource-surveys/streamcat-dataset) (Hill et al. 2016). To access the database, a WATERSKMZ kml file (https://www.epa.gov/waterdata) was downloaded into Google Earth. This interface allowed access to the upstream land cover data of each sampled stream segment. Since habitat values were assessed by the USEPA in 2006 and 2011, all samples collected before 2011 used the 2006 data set, and samples during and after 2011 used the 2011 data set. Thus, habitat was assessed within 12 years of caddisfly sampling for all streams, and ~75% of streams were assessed within 5 years. The land cover categories of forest, grassland, or wetland were categorized as “undisturbed”, whereas row crops, pasture, mining, or developed were categorized as “disturbed”. This land use categorization, while coarse, has been found to be an effective predictor of changes in overall caddisfly assemblages throughout our study region (Houghton and DeWalt 2021) and may be broadly applicable to other regions of the world where agriculture and urban development are the primary anthropogenic land uses.

Adult caddisflies were sampled using an ultraviolet light trap, which consisted of an 8-Watt portable ultraviolet light placed over a white pan filled with ~70% EtOH. Traps were placed adjacent to aquatic habitats at dusk and retrieved ~2 h later (Wright et al. 2013; Brakel et al. 2015; Fabian et al. 2024). Samples were collected only if the peak daytime temperature was >25 °C, the dusk temperature was >15 °C, and there was no noticeable wind or precipitation at dusk (Houghton 2004). Since both male and female aquatic insects collected with an ultraviolet light trap placed within 20–40 m of a habitat accurately reflect the assemblage of that habitat (Sode and Wiberg-Larson 1993; Peterson et al.1999; Sommerhäuser et al.1999; Brakel et al. 2015; Pereira et al. 2024), dispersal of adults between sites, while certainly possible, was considered unimportant.

Caddisfly sampling occurred based in part on accumulated degree-days (ADD) and ranged from late May in the southern portion of the study area to early August in the northern portion (Houghton and DeWalt 2021). Over 95% of samples were collected during June and July. Almost 80% of samples were taken within one standard deviation of the mean ADD value for the 903 samples. The outliers were nearly all from the northern extreme of the study area since ADDs in those regions don’t reach the values of the southern portion until mid- to late August, well after the observed peak flight period. Thus, samples from these regions had lower ADD values.

Collected specimens were identified to the species level using Houghton’s (2012) treatment of the Minnesota fauna or with various taxon-specific treatments as needed. Specimens were coded with their affinity for one of six different functional feeding groups (FFGs) based on Merritt et al. (2019): algal piercers, filtering collectors, gathering collectors, predators, scrapers, and shredders. Codes consisted of ‘0’ for no affinity for an FFG, ‘1’ low affinity, ‘2’ moderate affinity, ‘3’ high affinity, and ‘4’ near exclusive affinity (Chevene et al. 1994; Houghton 2021). These codes were converted to proportions: 0 = 0.0, 1 = 0.25, 2 = 0.50, 3 = 0.75, and 4 = 1.0, to multiply by determined ash-free dry mass (AFDM) values for each species (Beauchard et al. 2017). This approach more accurately reflected the feeding plasticity of aquatic insects than pure categorization (Dolédec et al. 2000; Gayraud et al. 2003).

AFDM values for each species were taken from Houghton and Lardner’s (2020) determination of 63 common caddisflies of the northcentral US. Species without a determined value were assigned the value of a congener of similar size. While this approach did not reflect differences in body size due to sexual dimorphism, specific habitat, larval food quality, or emergence timing, among other factors (Svensson 1975; Wagner 2002, 2005), it still allowed for a more precise determination of differences between sites than simply counting specimens and treating them as ecologically equivalent regardless of size (Houghton and Lardner 2020; Venarsky et al. 2020), while simultaneously allowing preservation of the vast majority of specimens as vouchers. Specimens have been deposited primarily in the Hillsdale College Insect Collection (HCIC), the Illinois Natural History Survey (INHS), and the University of Minnesota Insect Museum (UMSP).

From the above data, the caddisfly assemblage of each sample was compiled into 31 common water quality metrics within six categories and reflected three different levels of taxonomic resolution where appropriate (Table 1). Overall summary metrics included the total number of specimens, total organic (AFDM) biomass, and mean biomass per specimen. Taxonomic richness was determined at the family, genus, and species levels. The Hilsenhoff Biotic Index (HBI) (Hilsenhoff 1987; Barbour et al. 1999) was determined at the genus level, whereas the Biological Monitoring Working Party (BMWP) (Hawkes 1998) was determined at the family level. The Shannon diversity index (H’) was calculated at family, genus, and species levels as:

$H^{'}=-\sum_{i=1}^{\infty }pi(lnpi)$

The Simpson diversity index (D) was also calculated at family, genus, and species levels as:

$D=1-\sum_{i=1}^{\infty }pi^{2}$

**Table 1.** Summary statistics of 31 simple linear regression models produced from metrics calculated from adult caddisfly data and their ability to predict the known percentage of undisturbed habitat upstream of each of the 903 sampling sites of this study. HBI: Hilsenhoff Biotic Index. BMWP: Biological Monitoring Working Party.

Number	Metric	Coefficient	*P*	*R* ^2^
1	Number of specimens	0.01	<0.001	0.03
2	Total biomass	0.02	<0.001	0.05
3	Mean biomass per specimen	23.23	<0.001	0.04
4	Number of families	7.81	<0.001	0.59
5	Number of genera	3.52	<0.001	0.52
6	Number of species	1.72	<0.001	0.40
7	HBI value	-14.54	<0.001	0.37
8	BMWP value	0.42	<0.001	0.17
9	Shannon family diversity	39.75	<0.001	0.36
10	Shannon genus diversity	31.17	<0.001	0.27
11	Shannon species diversity	24.27	<0.001	0.22
12	Simpson family diversity	12.30	<0.001	0.25
13	Simpson genus diversity	6.24	<0.001	0.18
14	Simpson species diversity	3.69	<0.001	0.13
15	Percent dominant family	-77.80	<0.001	0.20
16	Percent dominant genus	-65.98	<0.001	0.13
17	Percent dominant species	-60.14	<0.001	0.12
18	Relative algal piercer biomass	-137.32	0.009	0.01
19	Relative gathering collector biomass	2.86	0.560	0.00
20	Relative filtering collector biomass	-59.77	<0.001	0.33
21	Relative predator biomass	29.44	<0.001	0.02
22	Relative shredder biomass	66.11	<0.001	0.27
23	Relative scraper biomass	145.23	<0.001	0.09
24	Shredder to filtering collector ratio	0.83	<0.001	0.10
25	Relative algal piercer specimens	-8.68	0.285	0.00
26	Relative gathering collector specimens	20.76	<0.001	0.02
27	Relative filtering collector specimens	-57.56	<0.001	0.28
28	Relative predator specimens	51.22	<0.001	0.05
29	Relative scraper specimens	119.62	<0.001	0.12
30	Relative shredder specimens	85.99	<0.001	0.18
31	Shredder to filtering collector ratio	93.08	<0.001	0.17

For both indices:

$pi=\frac{ni}{N}$

where *n* was the number of specimens within the *i*th taxon of a sample, while *N* was the total number of all specimens of a sample. Diversity indices transformed into Hill numbers (Chao et al 2014) were not included in the final analysis, since the Hill number metrics for both indices had slightly lower *R*^2^ values than the untransformed metrics. The percentage of sample AFDM dominated by the most abundant taxon was determined at the family, genus, and species levels. Finally, the relative percentage of each sample composed of each of the six FFGs was determined for both simple specimen counts and AFDM, as was the ratio of shredders to filtering collectors. The last metric was calculated as:

$\frac{\text{ shredders }}{\text{ filtering collectors }+1}$

for both specimens and biomass in order to account for filtering collector values of zero. The overall approach was to test as many common metrics as possible.

Each of the 31 metrics was individually tested for its ability to predict the known percentage of undisturbed habitat upstream of each sampling site using simple linear regression modeling. All 31 metrics were also tested for their collective ability to predict undisturbed upstream habitat using multiple linear regression modeling. Individual metrics from the multiple linear regression were evaluated for their individual importance to the overall model by calculated significance values and variance inflation factors (VIF). A final multiple linear regression was then calculated including only significant metrics with low VIF values. All statistical analyses were conducted using Excel for Windows with the Real Statistics add-in (www.real-statistics.com).

## ﻿Results

**Figure 2.**
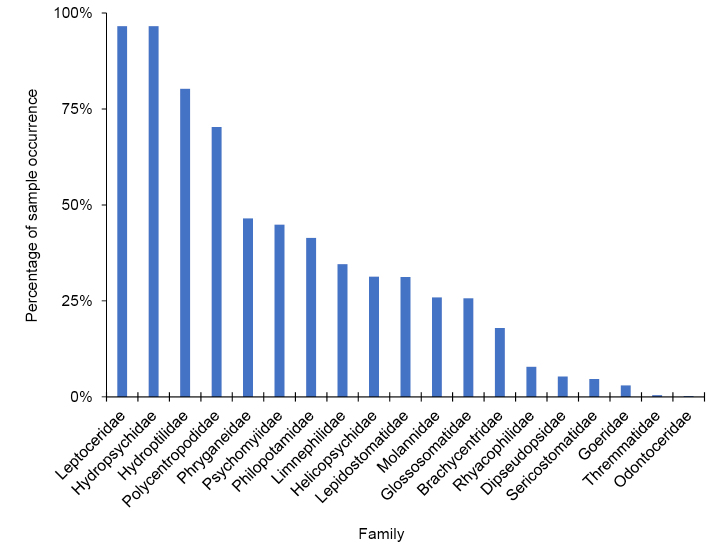
The percentage of the 903 samples that contained at least one specimen of the 19 caddisfly families collected during this study.

**Figure 3.**
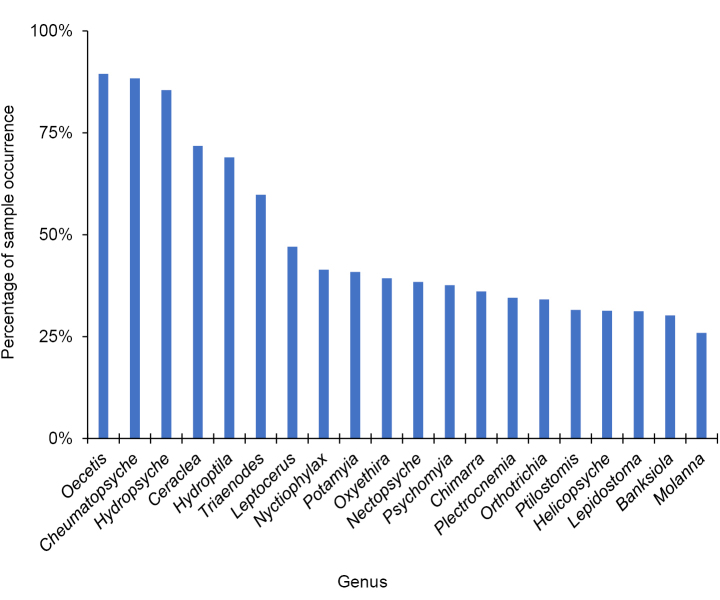
The percentage of the 903 samples that contained at least one specimen of the 20 most abundant caddisfly genera collected during this study.

**Figure 4.**
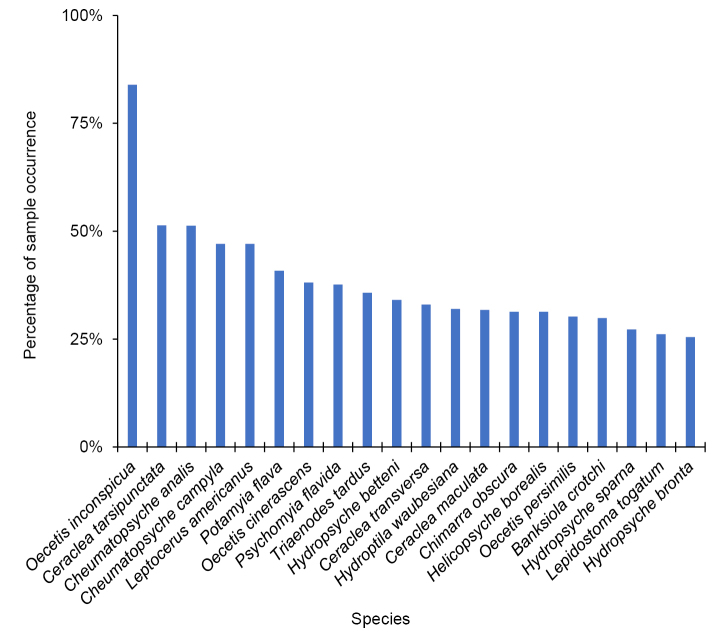
The percentage of the 903 samples that contained at least one specimen of the 20 most abundant caddisfly species collected during this study.

A total of 514,989 caddisfly specimens were collected and identified during this study, representing 299 species, 70 genera, and 19 families. The families Hydropsychidae and Leptoceridae were both collected in nearly 97% of samples, and Hydroptilidae and Polycentropodidae in 80% and 70%, respectively (Fig. 2). The most common genera were *Oecetis* (Leptoceridae) (90% of samples), *Cheumatopsyche* (Hydropsychidae) (88%), and *Hydropsyche* (Hydropsychidae) (85%) (Fig. 3). The most common species, by far, was *Oecetis inconspicua* (84%), followed by *Ceraclea tarsipunctata* (Leptoceridae) (52%) and *Cheumatopsyche analis* (51%) (Fig. 4).

**Figure 5.**
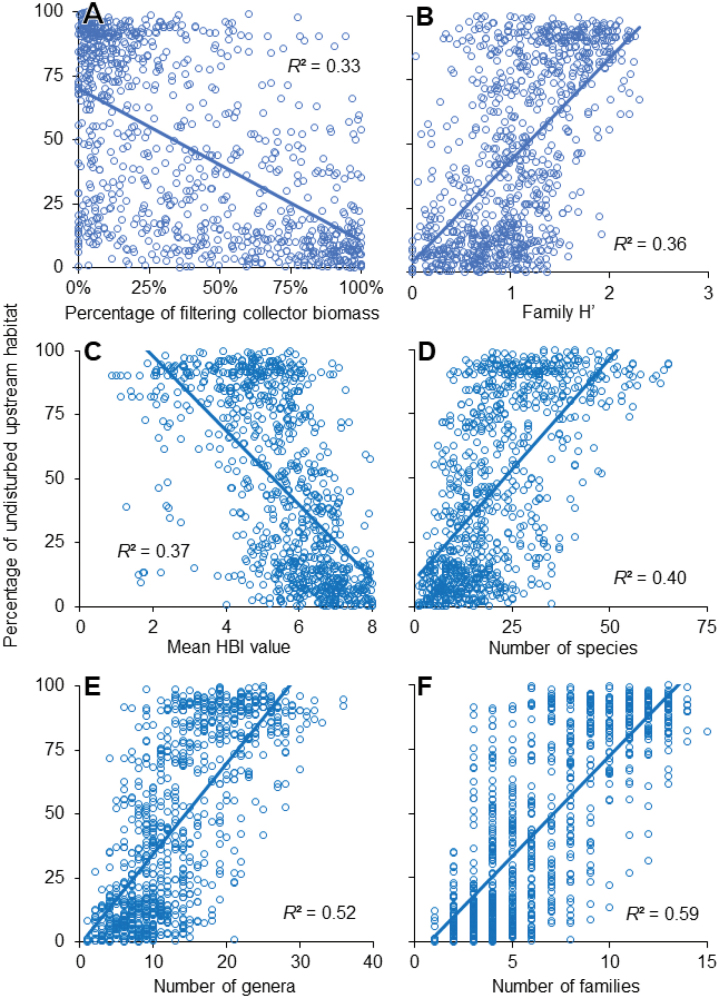
The six calculated metrics with the highest ability to predict the known percentage of undisturbed habitat upstream of each of the 903 sampling sites based on the simple linear regression models of Table 1. **A** percentage of filtering collector biomass (AFDM) (*y* = -59.77*x* + 70.03) **B** Shannon diversity index (H’) at the family level (*y* = 39.75*x* + 4.20) **C** Hilsenhoff Biotic Index (HBI) (*y* = -14.54*x* + 127.00) **D** number of species (*y* = 1.72*x* + 10.67) **E** number of genera (*y* = 3.52*x* - 0.86) **F** number of families (*y* = 7.81*x* - 5.44).

**Figure 6.**
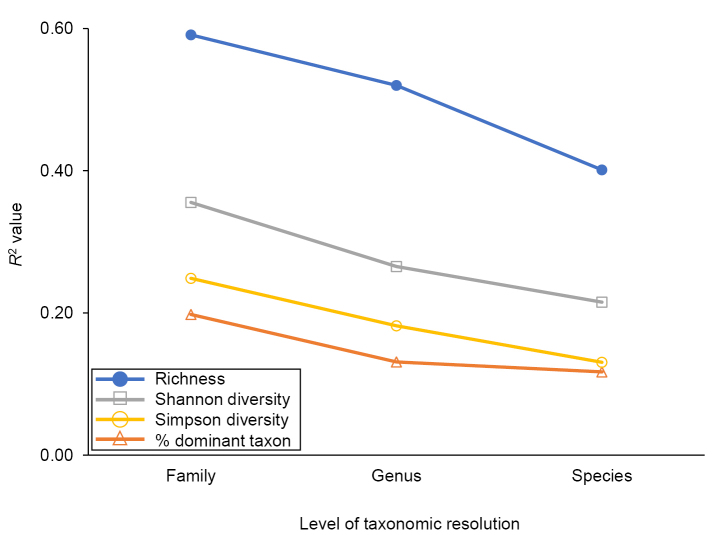
Coefficient of determination (*R*^2^) values for simple linear regression models produced using four different metrics at three different levels of taxonomic resolution. Data from Table 1.

Of the 31 simple linear regression models tested using adult caddisfly metrics, 29 were significant predictors of known undisturbed upstream habitat (Table 1), and six had *R*^2^ values >0.30 (Fig. 5). Shannon diversity at the family level (0.36) and taxonomic richness at the species (0.40), genus (0.52), and family (0.59) levels predicted an increase in undisturbed upstream habitat. Conversely, the percentage of filtering collector biomass (0.33) and the Hilsenhoff Biotic Index (HBI) (0.37) predicted a decrease. Of the four metrics tested at the family, genus, and species levels of taxonomic resolution, models using the family level had the highest *R*^2^ value in every case (Fig. 6).

A multiple linear regression analysis combining all 31 adult caddisfly metrics produced a significant model (*R*^2^ = 0.65) with nine significant metrics (Table 2). Due to their high (>10) VIF values, however, six of the metrics were removed. A multiple linear regression analysis using the metrics of family richness, HBI, and the ratio of shredders to filtering collectors produced a significant model (*R*^2^ = 0.62) with all three metrics retained as both significant and having a low VIF (Table 3).

**Table 2.** Results of a multiple linear regression model of the combined ability of all 31 metrics to predict the known percentage of undisturbed habitat upstream of each of the 903 sampling sites of this study. Overall model *R*^2^ = 0.65, *P* < 0.001. VIF: variance inflation factor. Metric numbers correspond to Table 1.

Number	Coefficient	SE	*T* statistic	*P*	VIF
Intercept	107.84	23.47	4.59	0.000	–
4	3.68	0.73	5.04	0.000	13.39
7	-4.93	1.03	-4.79	0.000	4.84
20	-68.65	22.13	-3.10	0.002	-9.01E+14
29	-48.48	18.04	-2.69	0.007	1.39+E14
19	-57.52	21.62	-2.66	0.008	4.29E+14
24	0.17	0.07	2.57	0.010	1.63
8	0.13	0.05	2.48	0.013	21.70
21	-52.40	23.09	-2.27	0.023	2.64E+15
18	-118.84	55.63	-2.14	0.033	1.29E+15
17	-22.67	13.52	-1.68	0.094	15.46
2	0.01	0.00	1.65	0.100	6.69
22	-33.79	20.84	-1.62	0.105	1.13E+15
12	-2.88	1.79	-1.61	0.108	13.59
9	12.23	7.63	1.60	0.109	33.94
30	-72.90	52.04	-1.40	0.162	1.50E+15
3	4.51	3.61	1.25	0.212	2.34
15	10.87	8.79	1.24	0.217	6.56
11	-8.59	8.10	-1.06	0.289	62.34
1	0.00	0.00	-1.01	0.312	7.17
6	-0.18	0.23	-0.81	0.421	18.15
28	11.30	15.00	0.75	0.451	5.15E+14
31	30.93	51.29	0.60	0.547	136.79
26	-6.41	10.89	-0.59	0.556	-4.50E+15
14	0.44	0.91	0.48	0.629	20.89
5	0.32	0.66	0.48	0.633	47.42
10	-3.73	10.71	-0.35	0.727	81.22
25	-3.26	11.19	-0.29	0.771	1.80E+15
13	0.42	1.60	0.26	0.794	31.19
16	1.93	15.27	0.13	0.899	18.30
23	0.00	0.00	0.00	0.000	-6.43E+14
27	0.00	0.00	0.00	0.000	-5.00E+14

**Table 3.** Results of a multiple linear regression of the combined ability of three metrics to predict the known level of undisturbed habitat upstream of each of the 903 sampling sites of this study. Overall model *R*^2^ = 0.62, *P* < 0.001. VIF: variance inflation factor. Metric numbers correspond to Table 1.

Number	Coefficient	SE	*T* statistic	*P*	VIF
Intercept	17.84	5.37	3.33	0.000918	–
4	6.61	0.29	22.87	1.34E-91	1.90
7	-3.08	0.69	-4.47	8.8E-06	1.96
24	0.31	0.06	5.43	7.12E-08	1.10

## ﻿Discussion

While linear regression models produced with nearly all of the metrics were statistically significant, the null hypothesis of *slope = 0* can easily be rejected by weak biological relationships, especially for large sample sizes (Cook and Weisberg 2009). Since most of the models had *R*^2^ values <0.30, their corresponding metrics were suboptimal predictors of undisturbed habitat. The weakest metrics were, not surprisingly, the relative abundance of generalist FFGs, such as gathering collectors, predators, and algal piercers, as well as the number and biomass of specimens. Such metrics are rarely used for biomonitoring with benthic macroinvertebrates and are probably of negligible value for adult caddisfly biomonitoring as well.

The strongest models exhibited expected relationships between metric values and the percentage of undisturbed upstream habitat (Fig. 5). The observed increase in undisturbed upstream habitat predicted by both increased taxonomic richness and increased diversity indices at all taxonomic levels has been previously reported across many taxa, geographic regions, and disturbance types (Murphy and Romanuk 2014), including adult caddisflies (Houghton and DeWalt 2023). The HBI, which was first developed in the Upper Midwest ecoregion of this study, assigns taxa assumed to be tolerant of organic pollution higher values; thus, decreasing HBI values predicted an increasing percentage of undisturbed upstream habitat (Hilsenhoff 1987; Barbour et al. 1999). Since species in the shredder FFG are directly dependent on the input of their coarse allochthonous food source, it was expected that their increasing relative abundance would predict increasing levels of undisturbed habitat (Houghton et al. 2011; Dohet et al. 2014; Entrekin et al. 2020; Houghton 2021; Williams and Houghton 2024). Conversely, species in the filtering collector FFG tend to be some of the most tolerant of aquatic insects, feed on fine particulate organic matter, and can increase in polluted streams; thus, their increasing relative abundance predicted decreasing undisturbed habitat (Houghton 2007; Hawkins and Yuan 2016; Kaelin and Altermatt 2016; Houghton and DeWalt 2021).

**Figure 7.**
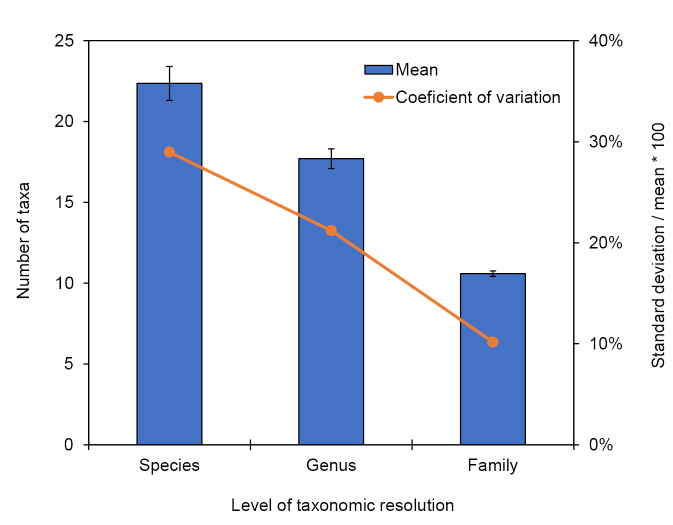
Mean (±SE) number of taxa caught from 38 summer ultraviolet blacklight samples of a first-order stream in northern Michigan (primary axis), along with the coefficient of variation for each level of taxonomic resolution (secondary axis). Raw data from Houghton (2018).

The best predictive models were all produced using simple taxonomic richness data. Somewhat surprisingly, family richness produced a better model than genus, and genus a better model than species. Discussions about the appropriate taxonomic resolution for biomonitoring protocols have been ongoing for several decades, with most researchers positing that species-level resolution is ideal when possible due to an increase in total information, precision, sensitivity, and accuracy of metrics (Jones 2008; Resh 2023). A potential counterpoint to this argument is the greater stochastic variation inherent in species-level datasets relative to those of broader taxonomic resolution. For example, Houghton (2018) sampled a single first-order stream in northern Michigan (USA) (44°02'52"N, 85°39'22"W) approximately weekly for several years using the same protocols and weather parameters of the current study, obtaining 38 samples during the peak summer sampling period. Within that data set, the family richness coefficient of variation was <1/2 the value of genus richness variation, and ~1/3 the value of species richness variation (Fig. 7). A similar phenomenon probably occurred during the current study, as the capture of a species will always affect species richness, but may not affect family or genus richness, particularly that of taxa that contain many species. Other metrics calculated using taxonomic data, such as diversity indices and percent dominant taxon, would similarly be affected by this higher variation among species (Fig. 6).

The behavior of caddisfly adults is poorly known, and much of this unaccounted species variation is probably from subtle differences in the emergence phenologies, flight periodicities, and adult behavior of species at specific locations. Local variables, such as water temperature, nutrient input, or other aspects of water physicochemistry may affect specific emergence timing, which would affect species richness between different dates. While nearly all Nearctic species are attracted to ultraviolet lights, it is not definitively known if ultraviolet light trapping is a truly exhaustive technique for sampling caddisflies (Myers and Resh 1999; Nakano and Tanida 1999), if it attracts all species equally, if species may be more attracted at certain points during their adult flight period, or if factors such as larval food quality or general health of specimens may affect attraction. Further, while the low vagility of caddisflies limits “leakage” between habitats (Sode and Wiberg-Larson 1993; Peterson et al. 1999; Sommerhäuser et al. 1999; Calor and Mariano 2012; Pereira et al. 2024) especially between habitats that are not in view of each other (Brakel et al. 2015; Houghton and Haack 2023), such dispersals are still possible.

Results from the multiple linear regression analysis reinforced the value of family richness as a predictor of undisturbed upstream habitat since including all 31 metrics only increased *R*^2^ values from 0.59 to 0.65 over the simple linear regression model using family richness exclusively. Although nine of the 31 metrics in the multiple regression explained a significant amount of variation in undisturbed upstream habitat, seven had VIF values >10 and five had values >1×10^14^. While there is some debate about its importance and specific thresholds, VIF values >10 suggest high levels of collinearity with other variables and, thus, redundancy (O’Brien 2007; Marcoulides and Raykov 2019). The final model, therefore, only included family richness, HBI, and the ratio of shredders to filtering collectors. These metrics produced a model *R*^2^ value of 0.62, a result only marginally improved over the regression model using only family richness.

Further research is necessary to quantify differences in emergence phenology, flight periodicities, and potential light attraction variability in order to decrease the stochastic variation found at finer taxonomic levels. If such variation can be added to models, then species richness may be more predictive of undisturbed habitat than that of family or genus. In addition, other ways of quantifying habitat disturbance should be tested to determine if adult caddisfly metrics are successful at predicting differences in physicochemistry or other aspects of water pollution in addition to coarse determinations of habitat disturbance.

While it is not the objective of this paper to definitively resolve debates about taxonomic resolution or the most effective life stage or taxon for freshwater biomonitoring, it is worthwhile to note that family-level richness using adult caddisflies – a very simple and basic metric – was successful at explaining nearly 60% of the variation in habitat disturbance upstream of sampling sites over a large geographic area. This metric eliminates the microhabitat sampling bias inherent in all benthic studies, requires only an ultraviolet blacklight trap, and can be implemented in both the lab and the field with minimal training or expertise. Clearly, adult caddisflies have value as a biomonitoring taxon, and family richness may be a particularly effective metric due to low stochastic variation and ease of use.

## ﻿Additional information

### Author ORCIDs

David C. Houghton https://orcid.org/0000-0001-6946-4864

### Data availability

Data will be made available upon reasonable request.
